# Development of an intraoperative breast cancer margin assessment method using quantitative fluorescence measurements

**DOI:** 10.1038/s41598-022-12614-6

**Published:** 2022-05-20

**Authors:** Hiroki Ueo, Itsushi Minoura, Hiroaki Ueo, Ayako Gamachi, Yuichiro Kai, Yoko Kubota, Takako Doi, Miki Yamaguchi, Toshinari Yamashita, Hitoshi Tsuda, Takuya Moriya, Rin Yamaguchi, Yuji Kozuka, Takeshi Sasaki, Takaaki Masuda, Yasuteru Urano, Masaki Mori, Koshi Mimori

**Affiliations:** 1 Ueo Breast Cancer Hospital, 1-3-5 Futamatamachi, Oita, 870-0887 Japan; 2 Department of Surgery and Sciences, Graduate School of Medical Sciences, Kyushu University, Fukuoka, 812-8582 Japan; 3 Goryo Chemical Inc., Kita 8 Nishi 18-35-100, Chuo-ku, Sapporo, 060-0008 Japan; 4 Department of Pathology, Almeida Memorial Hospital, Oita, 870-1195 Japan; 5 Breast Cancer Center, Shonan Memorial Hospital, Kamakura, 248-0027 Japan; 6 Department of Breast Surgery, JCHO Kurume General Hospital, Kurume, 830-0013 Japan; 7 Department of Breast and Endocrine Surgery, Kanagawa Cancer Center, Yokohama, 241-8515 Japan; 8 Department of Basic Pathology, National Defense Medical College, Tokorozawa, 359-8513 Japan; 9 Department of Pathology, Kawasaki Medical School, Kurashiki, 701-0192 Japan; 10 Department of Pathology and Laboratory Medicine, Kurume University Medical Center, Kurume, 839-0863 Japan; 11 Department of Pathology, Mie University Hospital, Tsu, 514-8507 Japan; 12 Department of Next-Generation Pathology Information and Networking, Faculty of Medicine, The University of Tokyo, Tokyo, 113-0033 Japan; 13 Department of Surgery, Kyushu University Beppu Hospital, Beppu, 874-0838 Japan; 14 Graduate School of Medicine and Graduate School of Pharmaceutical Sciences, The University of Tokyo, Tokyo, 113-0033 Japan

**Keywords:** Cancer imaging, Diagnostic markers

## Abstract

Breast-conserving surgery has become the preferred treatment method for breast cancer. Surgical margin assessment is performed during surgery, as it can reduce local recurrence in the preserved breast. Development of reliable and lower-cost ex vivo cancer detection methods would offer several benefits for patient care. Here, a practical and quantitative evaluation method for the ex vivo fluorescent diagnosis of breast lesions was developed and confirmed through a three-step clinical study. Gamma-glutamyl-hydroxymethyl rhodamine green (gGlu-HMRG) has been reported to generate fluorescence in breast lesions. Using this probe, we constructed a reliable and reproducible procedure for the quantitative evaluation of fluorescence levels. We evaluated the reliability of the method by considering reproducibility, temperature sensitivity, and the effects of other clinicopathological factors. The results suggest that the fluorescence increase of gGlu-HMRG is a good indicator of the malignancy of breast lesions. However, the distributions overlapped. A 5 min reaction with this probe could be used to distinguish at least part of the normal breast tissue. This method did not affect the final pathological examination. In summary, our results indicate that the methods developed in this study may serve as a feasible intraoperative negative-margin assessment tool during breast-conserving surgery.

## Introduction

Breast cancer is one of the most frequent cancers in women^1^, and breast-conserving surgery (BCS) followed by radiation therapy has become the preferred treatment method^2,3^. Intraoperative assessment of the surgical margin is performed, as it can prevent reoperation and local recurrence in the preserved breast^4–6^. Pathological evaluation with intraoperative frozen section analysis (IFSA) is a reliable method of achieving a clear surgical margin^7–9^. However, IFSA is time-consuming and requires human resources, such as the involvement of pathologists, as well as cost and space to set up the instruments^10^. Because of the inadequate number of available pathologists^11^, only a limited number of samples is examined with IFSA in many institutes^10^, leading to an increase in false-negative results. Therefore, ex vivo margin assessment could be replaced with an alternative technique with satisfactory sensitivity and specificity provided at a reasonable cost.

To this end, techniques used to intraoperatively detect cancerous lesions in vivo using fluorescent probes have been developed, approved, and clinically used^12–15^. Furthermore, various fluorescent probes are under development^16–18^. Most of them are injected or topically sprayed to produce fluorescence and enable determination of cancerous lesions. The usefulness of these techniques depends on the accuracy of distinguishing between lesions that should be resected and those that do not require resection.

For ductal carcinoma in situ (DCIS), breast-conserving surgery followed by radiation therapy is the standard treatment, since some DCIS cases will likely progress to invasive cancer. However, different views on the evaluation and resection criteria for breast lesions have been reported. For example, it was reported that breast cancer-specific survival was identical between patients with low-grade DCIS who did or did not undergo surgery^19,20^. Therefore, cancer-detecting fluorescent probes may be expected to facilitate the detection of malignant lesions under the present diagnostic guidelines and also enable the evaluation of the malignancy of lesions with quantitative marker assessment. Unfortunately, intraoperative evaluation of the fluorescence intensity in vivo is not practical because it is not easy to precisely adjust the excitation light intensity, probe concentration, and distance from a lesion to the optical system, all of which are required for accurate quantitative evaluation.

For this purpose, ex vivo quantitative evaluation of fluorescence intensity of surgically resected cancerous lesions is practical to measure the amount of a marker protein or enzyme activity. In addition, ex vivo detection is more accessible and can be achieved at a lower cost. To establish the reliability of such a technique, we collected fundamental data for quick and pragmatic ex vivo detection of cancer using a fluorescent probe.

In this study, we report the fundamental data used to establish a reliable and practical technique for ex vivo fluorescence detection. This method was applied in a multicenter study to test the performance of gamma-glutamyl-hydroxymethyl rhodamine green (gGlu-HMRG), a chemical probe that detects gamma-glutamyltransferase (GGT) activity^21–23^, to detect breast lesions. We recently reported the major results of a multicenter study as a short letter^24^, where we determined the negative threshold of the 5 min fluorescence increase (5 min FI), which indicates negative margins. We reanalyzed the data to explore the possibility of evaluating the malignancy of the lesion, considering the FI of low-grade DCIS. In addition, we report our re-validation of the results after measuring 46 additional samples to confirm our previous findings.

## Results

### Improvement of the assay protocol

**Figure 1:**
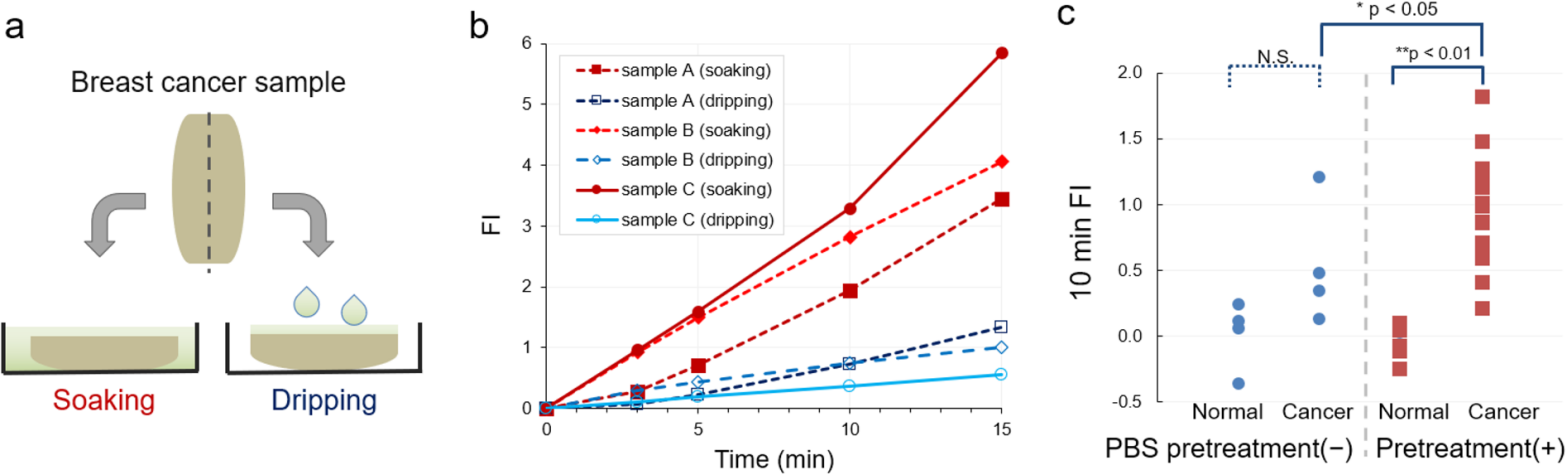
Comparison of the fluorescence measurement procedures tested in this study*.* **(a**) Schematic diagram of the testing method used to compare the new soaking and the previous dripping procedures. (**b**) Three representative examples showing differences between the new soaking and the previous dripping procedures. Filled symbols with red lines indicate the FIs of samples that were soaked in the probe solution. Open symbols with blue or cyan lines indicate the FIs of samples onto which sample solutions were dripped before the measurements. (**c**) FIs with or without pre-soaking in PBS solution for > 0.5 min. *p*-values were calculated using the Steel–Dwass test. N.S. indicates not significant (*p* > 0.05).

First, we tested the reproducibility of previous results^22,23^. Unfortunately, we observed many false-negative results in the first trial, including cancer tissues that only showed a slight fluorescence increase (FI). With these false negatives, the cancer tissues appeared hard and raised compared to the relatively soft surrounding tissue. Therefore, after dripping and spraying the probe solution, it flowed down from the top surface, without penetrating the tissue. To avoid false negatives, we adopted an improved protocol with pre-soaking with phosphate-buffered saline (PBS) and then soaking the sample to keep providing enough amount of probe molecules during measurements. This new soaking protocol was compared with the previous dripping protocol by cutting the cancerous sample into two pieces and executing both methods simultaneously (Fig. 1a). This improved protocol resulted in a higher FI (Fig. 1b). In addition, the effect of pre-soaking the tissues with PBS was tested. This procedure resulted in a significantly higher FI in cancerous tissues (Fig. 1c). In addition, PBS pre-soaking for more than 20 min resulted in slightly higher FI for both cancerous and normal tissues (Fig. S1). Therefore, we adopted the improved protocol that included soaking the sample tissues in gGlu-HMRG solution after soaking them in PBS for 0.5–20 min, in the following measurements.

**Figure 2:**
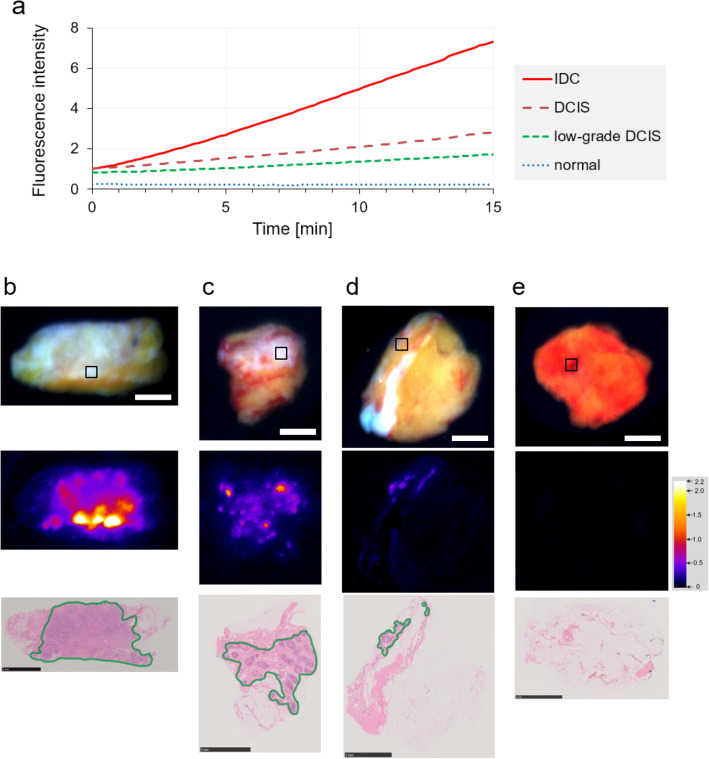
Representative examples of fluorescence intensity changes*.* A representative example of each IDC, DCIS, low-grade DCIS, and normal breast tissue (N = 1) is indicated. (**a**) Fluorescence intensity changes in the areas shown by the squares in the visible light images in panels (**b**–**e**). (**b**–**e**) Visible light images, 5 min FI images with pseudocolor, and HE-stained images of various samples. The green lines indicate areas of malignant cells. (**b**) Invasive ductal carcinoma. (**c**) Middle/high-grade DCIS. (**d**) Low-grade DCIS. (**e**) Normal breast tissue, including normal mammary glands. The scale bars indicate 5 mm.

We started by establishing an FI-measurement protocol followed by measuring the time-dependent fluorescence intensity changes in various breast lesions. A representative example of each tissue type shown in Fig. 2 suggests that the FI values depended on the malignancy of the breast lesions. In some samples, lesions as small as 1–3 mm, that were detected as one or two pixels of fluorescent signals, could be detected (Fig. S2). In these small lesions, the FI also differs depending on the malignancy of the lesions. To further evaluate these differences, we accumulated FI data in a multicenter study.

### Comparing FIs between cancerous and normal tissues

In this multicenter study, we evaluated 309 samples from four institutes. Among them, 10 measurements did not precisely follow the approved procedure. In addition, for 48 specimens the categorization into four groups (invasive, non-invasive, proliferative lesions, and normal) by the four pathologists was not consistent. Therefore, we excluded the data for these 58 samples, and the FI values of 138 non-cancerous and 113 cancerous samples were analyzed.

**Figure 3:**
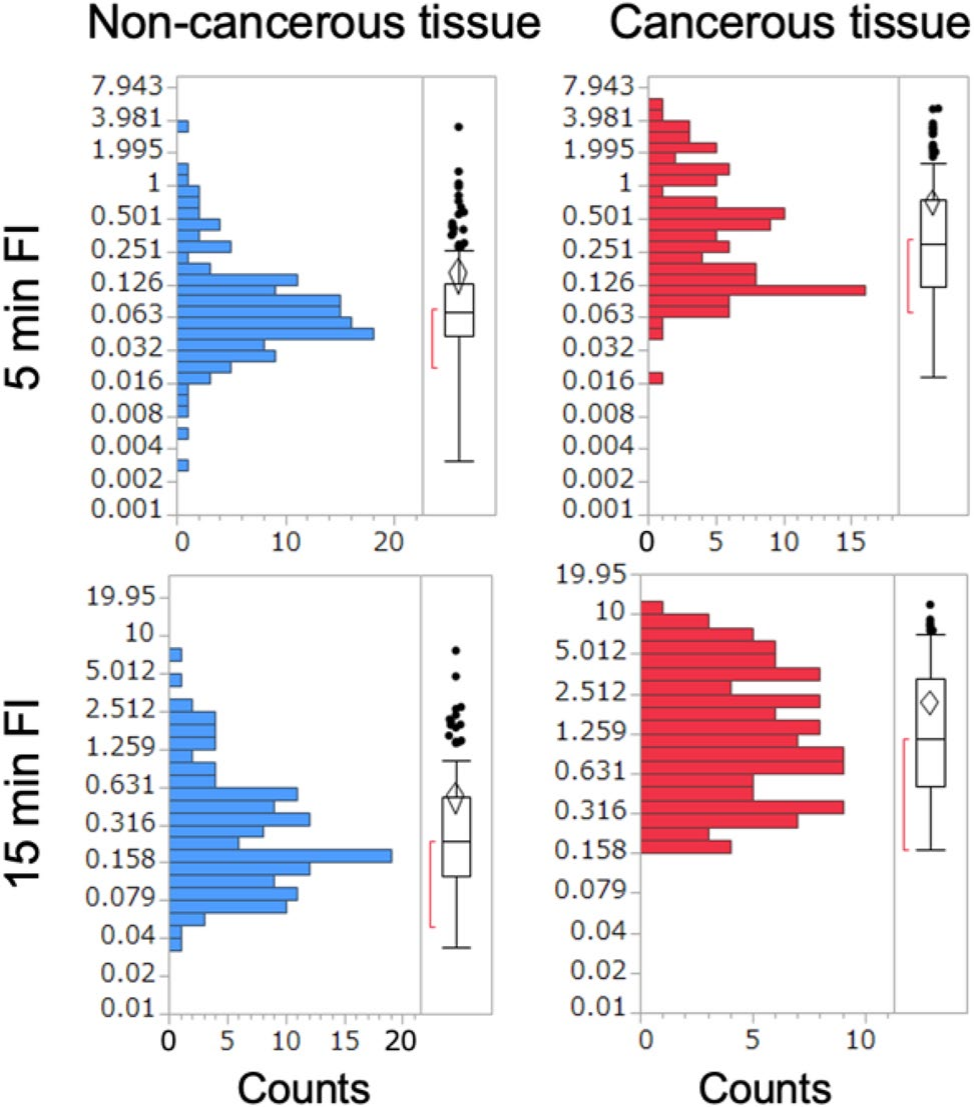
Distributions of 5 min and 15 min FIs of cancerous and non-cancerous tissues. The dataset obtained in the multicenter study, including 251 sample measurements (138 non-cancerous and 113 cancerous tissues), was analyzed. Distributions and statistical parameters are indicated by histograms and box-and-whisker plots. The FIs are shown on a logarithmic scale.

The 5 min and 15 min FIs of cancerous and non-cancerous tissues did not follow a normal distribution (Fig. S3). However, these values were unexpectedly closer to the log-normal distribution. Thus, by plotting the FI on a logarithmic scale, the distributions could be better distinguished (Fig. 3). This result suggests that GGT activity or expression in normal and cancer cells can be better described by log-normal distributions than by normal distributions. This result is consistent with previous theoretical and experimental studies those demonstrated that protein-expression and mRNA-expression levels in single cells follow a log-normal distribution because of a complex intracellular signaling network^25,26^. Therefore, we have presented the FI values on a log scale and applied nonparametric statistical tests.

The data revealed a significant difference in FIs between cancerous and non-cancerous tissues. The 5 min FI values of cancer tissues (0.72 ± 1.01; mean ± standard deviation [S.D.], N = 113) was significantly higher (*p* < 0.0001, Wilcoxon rank-sum test) than those of non-cancer tissues (0.15 ± 0.35, N = 138). Based on receiver-operating characteristic analyses, the areas under the curve were 0.836 and 0.835 for the 5 min and 15 min FIs, respectively (Fig. S4). These data suggests that a 5 min measurement is sufficient to evaluate the FI; therefore, 5 min FIs were mainly used in the following analyses.

### Correlation between temperature and other background information

Using the multicenter study data, we attempted to identify factors that influence the FI values. First, we evaluated the effects of temperature because enzyme activities are usually temperature dependent. Our measurements showed that beef-kidney-derived GGT activity increased linearly when the temperature was increased from 20 to 40 °C (Fig. S5). Considering that the FI is a measure of GGT enzyme activity, it is important to understand the effect of temperature on the FIs of clinical specimens. The 5 min FI did not show a clear association with the temperature (Fig. S6). Although a significant correlation between the 5 min FI and the temperature was not observed, significant correlations (*p* < 0.05) were observed between the 15 min FI and the temperature (Table 1). Nevertheless, the 95% confidence intervals (CIs) for the correlations were between 0.4 and − 0.4, indicating that the correlation was weak.

**Table 1:** Correlations between FIs of clinical samples and ambient temperature.

Sample type	Variable	Correlation	95% CI		*p*-value
Cancerous	5 min FI	0.165	− 0.021	0.339	0.081
	15 min FI	0.189	0.004	0.361	**0.045**
Non-cancerous	5 min FI	0.156	− 0.013	0.316	0.070
	15 min FI	0.182	0.014	0.340	**0.034**

FI, fluorescence increase; CI, confidence interval.

Significant values are in bold.

No significant difference in 5 min FI among the four breast cancer subtypes was detected (Table S1). Among the clinicopathological features studied, no significant differences were detected (Table S2).

Thus, temperature and other clinicopathological features of the tissues did not affect the 5 min FI.

In addition, we confirmed the reliability of the standardized protocol by comparing the 5 min FIs of cancerous samples among the four institutes. No significant differences in FIs were detected between the four institutes (*p* = 0.087, Kruskal–Wallis). Similar results were obtained by pairwise comparisons between each institute using the Steel–Dwass test (Table 2).

**Table 2:** Differences in the 5 min FIs of cancer samples among the four institutes.

Institute	Institute	Difference of mean	S.E. of the difference	Z	*p*-value	Hodges–Lehmann estimator	95% CI	
C	D	12.42	6.70	1.853	0.249	0.277	− 0.082	0.851
A	D	7.81	3.72	2.097	0.154	0.426	− 0.131	0.700
B	D	6.93	3.14	2.205	0.122	0.470	− 0.082	1.269
C	A	1.89	5.95	0.317	0.989	0.042	− 0.310	0.372
A	B	− 4.22	3.77	− 1.118	0.678	− 0.214	− 0.775	0.264
C	B	− 8.24	6.28	− 1.312	0.555	− 0.208	− 0.601	0.294

S.E., standard error; Z, Z-value of Steel–Dwass test; CI, confidence interval; FI, fluorescence increase.

### Quantitative differences in FIs between different lesion types

**Figure 4:**
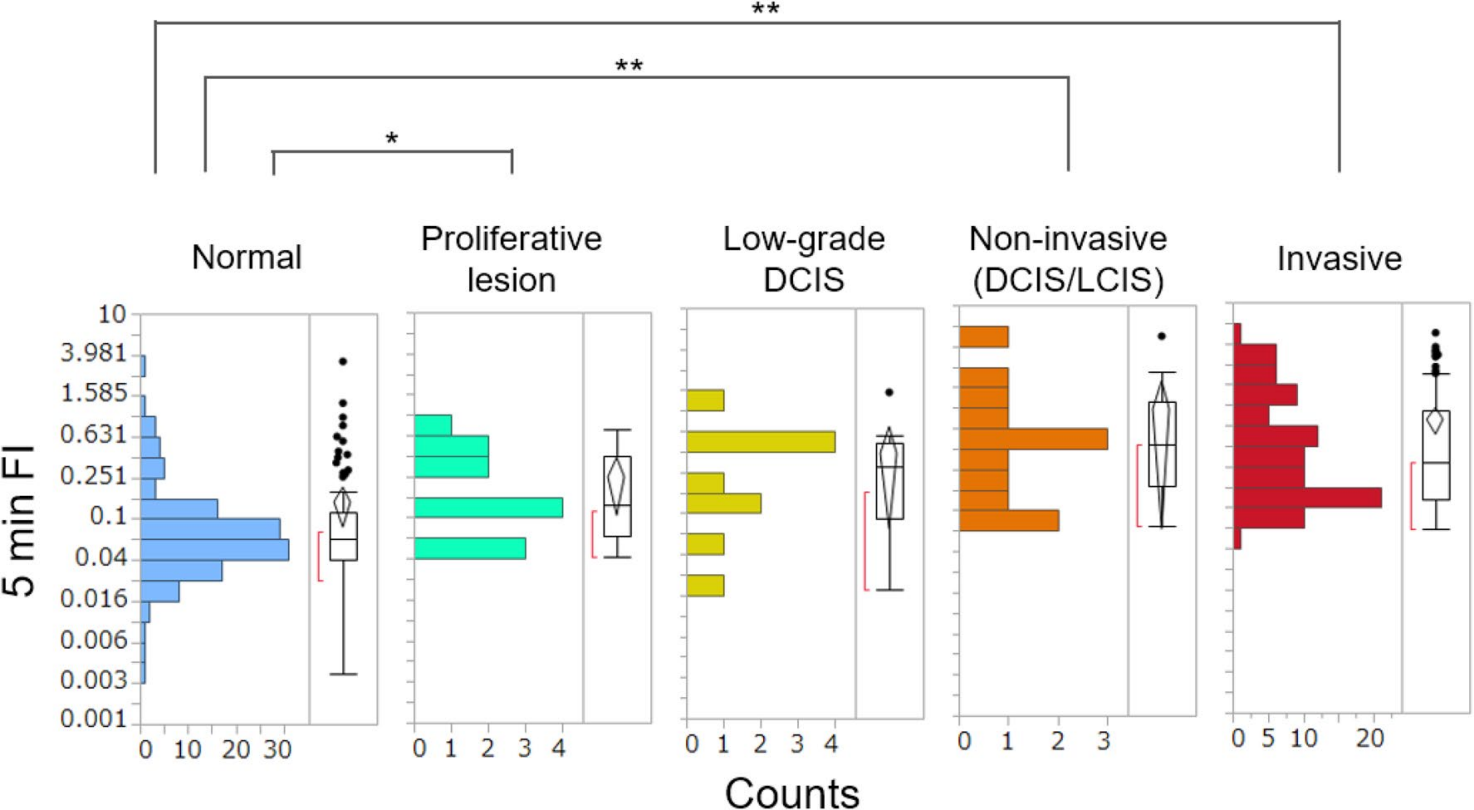
Differences in the 5 min FI distributions between breast cancer tissues of different lesion types*.* The dataset obtained in the multicenter study, including 251 sample measurements, was analyzed. *p < 0.05; **p < 0.001.

The distributions of the 5 min FIs between different tissue types, including invasive and non-invasive cancer, low-grade DCIS, proliferative lesions, and normal breast tissues are shown in Fig. 4 (see Table S3 for statistical parameters). The mean 5 min FIs increased with tissue malignancy. Spearman’s rank correlation coefficient was 0.60 (*p* < 0.001) for both the 5 min FIs and 15 min FIs. The malignancy was scored as 5 for invasive, 4 for non-invasive, 3 for low-grade DCIS, 2 for a proliferative lesion, and 1 for normal tissue. These data suggest that the 5 min FIs correlated with the malignancy of the lesions. Further, the 5 min FIs of invasive and non-invasive cancers (except for low-grade DCIS) and proliferative lesions were significantly larger than those of normal breast tissues (Table S4). However, a significant difference between low-grade DCIS and normal tissues was not detected (*p* = 0.066, Steel–Dwass test).

### Re-confirming the determined threshold

**Figure 5:**
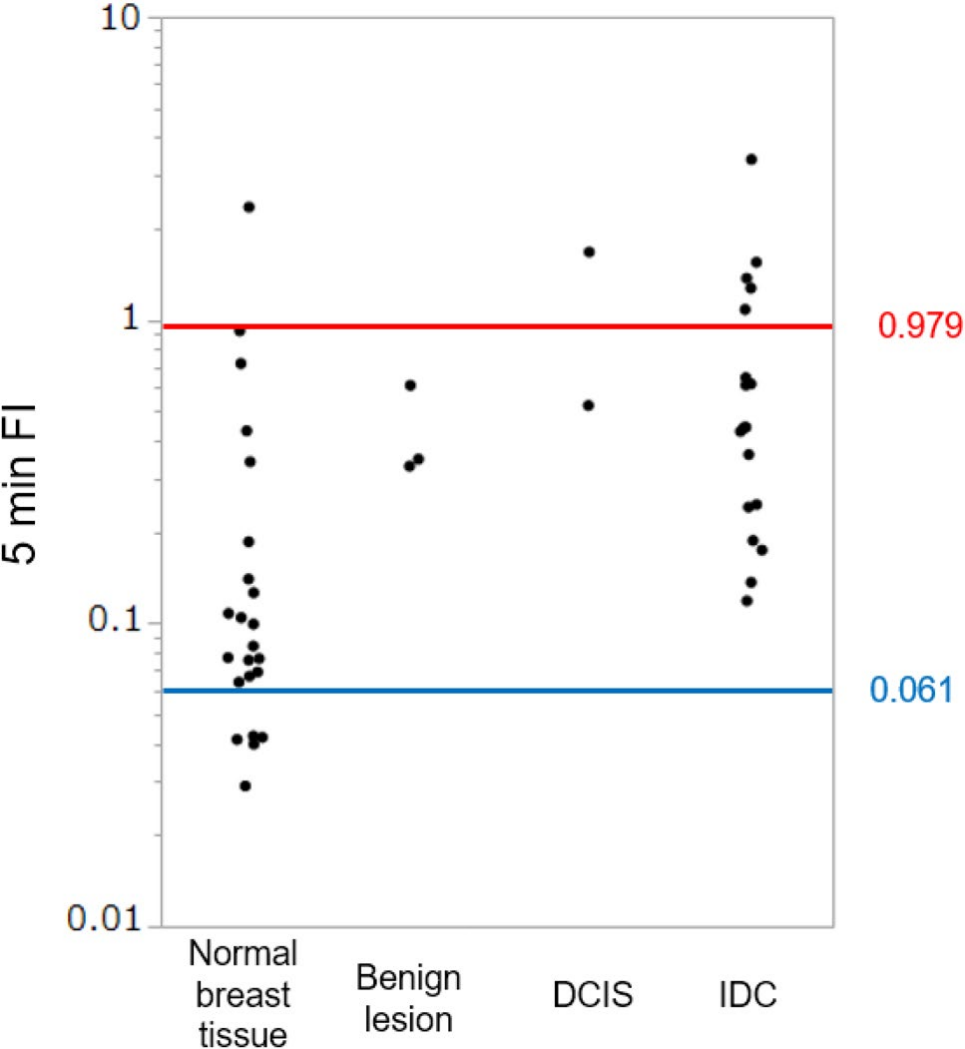
Re-validation of the threshold determined in the multicenter study data obtained from the validation study of 46 samples were plotted. The red (0.979) and blue (0.061) lines represent the positive and negative thresholds, respectively, as determined by the multicenter study that was reported previously^24^.

After the multicenter study, additional fluorescence measurements and pathological examinations were performed to re-evaluate the reliability of the obtained threshold. Among the 23 normal breast tissues, the 5 min FIs of five samples were below the negative threshold. All 20 malignant tissues showed a 5 min FI above the negative threshold. No false-negative results were obtained (Fig. 5, Table S6).

## Discussion

This study focused on the reliability of evaluating FIs with a chemical probe to detect differences between breast tissue features. For this purpose, we applied gGlu-HMRG, a chemical probe that can detect GGT enzyme activity via green fluorescence. Previously, we reported the detection of breast lesions and lymph node metastasis using this probe^22,23^. We established a procedure to obtain quantitatively reliable FI using a newly developed dedicated apparatus for fluorescence measurements. Unexpectedly, the fluorescence measurements using the previous method were not reproducible. In this study, we found that soaking the tissue entirely in the solution yielded better results than spraying or dripping the tissues. Using this method, a sufficient amount of probe molecules was provided to the cancer cells. In addition, rinsing with PBS improved the FIs of cancer tissues, probably because this step can remove blood, fat, and other materials that sometimes cover the cell surface (Fig. 1). In addition, rinsing with such a calcium-free solution may loosen cell–cell adhesion via cadherin molecules, and thus, facilitate probe binding with GGT, which is expressed on the cell surface^21^.

First, we developed a standardized protocol and applied it in a multicenter study. The temperature was predicted to have the greatest influence on the FI measurements. However, we did not need to precisely control the temperature when measurements were taken in the range of room temperature (15–30 °C), because the correlation between the temperature and 5 min FI was weak and not significant (P > 0.05, Table 1). This result does not necessarily deny the relationship between temperature and FI. Rejection of a statistical hypothesis is not a proof of ‘no relationship’ but only indicates that we failed to prove the relationship in the sample size. In our case, the distributions of FI (Fig. S6) suggest that the variation in GGT activity between cells and tissues was more significant than the effect of temperature, especially in 5 min FI. On the other hand, small but significant differences in the 15 min FI and temperature were observed (Table 1). This result could be explained by a hypothesis that 15 min FI became larger and as a consequence, the effect of temperature became more prominent. In this study, we chose to focus on 5 min FI and not to control the temperature to evaluate the feasibility of a simple, quick and cost-effective measurement method, however, controlling the temperature can still be considered for more accurate measurements.

The reliability of the established protocol was confirmed in both the multicenter and validation studies. In the multicenter study, no significant difference between institutes was observed (Table 2). This finding indicates that we could establish a protocol to detect FI with high reproducibility. The results of the validation study were also consistent. No cancer sample showed a 5 min FI below the negative threshold in this study (Fig. 5, Table S6).

Based on the data obtained from the 251 samples analyzed in the multicenter study, the 5 min FI of gGlu-HMRG appeared to correlate with the malignancy of the lesions. However, significant differences between proliferative lesions, low-grade DCIS, middle/high-grade DCIS, and invasive cancer were not detected. The distributions of the 5 min FIs of these lesions overlapped (Fig. 4). This result indicates that we could not distinguish these lesions using this probe, including differences between proliferative lesions and invasive cancers. Therefore, false positives could not be avoided. Surgeons must be careful not to perform unnecessary surgery. To establish a more reliable method for ex vivo diagnosis, the application of other chemical probes or their use in combination with other probes is required. Indeed, several new probes that can detect breast lesions have been recently developed^27,28^. The simple protocol we developed here could be applied to or used with these probes for a better and more cost-effective diagnosis.

Our three-step study confirmed that the negative threshold of the 5 min FI (0.061) could be used to verify the negative margins within 10 min. At least, we confirmed that no invasive cancer was left on the margin surface if the 5 min FI was below the threshold. In the multicenter study, two samples below the threshold contained low-grade DCIS. Thus, if a surgeon judged that the lesions could be controlled with radiotherapy or chemotherapy, then no additional surgery was required. Alternatively, by setting the negative threshold of the 15 min FI to 0.170, false negatives could be avoided for low-grade DCIS (Fig. S7).

Furthermore, our fluorescence-based diagnostic procedure did not prevent further pathological examination of the same sample. Analyzing pathological specimens with the procedure developed here may also help in determining postoperative therapy. Therefore, in combination with the improvement of probes, the method described here can provide reliable intraoperative navigation for surgeons.

## Methods

### Clinical samples

First, we examined samples from patients who underwent mastectomy or BCS (84 patients) at the Ueo Breast Cancer Hospital between 2016 and 2019 to improve the assay protocol. Then, we conducted a multicenter study in four institutes, including 108 patients who underwent surgery between August 2019 to March 2020. Overall, 309 samples were analyzed. Finally, based on the negative threshold designated in the previous multicenter study, additional validation was performed with 46 samples (18 invasive cancer, 2 non-invasive cancer, 3 benign/proliferative lesions, and 23 normal mammary tissues) from 19 patients treated at the Ueo Breast Cancer Hospital between February 2020 and September 2021.

Before sample acquisition, each patient provided written informed consent. The Ethics Review Committee of Osaka Chiken Hospital approved the study protocol at Ueo Breast Cancer Hospital. Ethics Review Committee of Almeida Memorial Hospital, Shonan Memorial Hospital, JCHO Kurume General Hospital, Kanagawa Cancer Center, National Defense Medical College, Kawasaki Medical School, Kurume University Medical Center, and Mie University Hospital approved the study protocol. All experimental methods were carried out in accordance with the approved protocols. The multicenter study was registered with the Japan Pharmaceutical Information Center Clinical Trials Information under identification number JapicCTI-195091 on December 25, 2019.

### Sample preparation and fluorescence measurements

**Figure 6:**
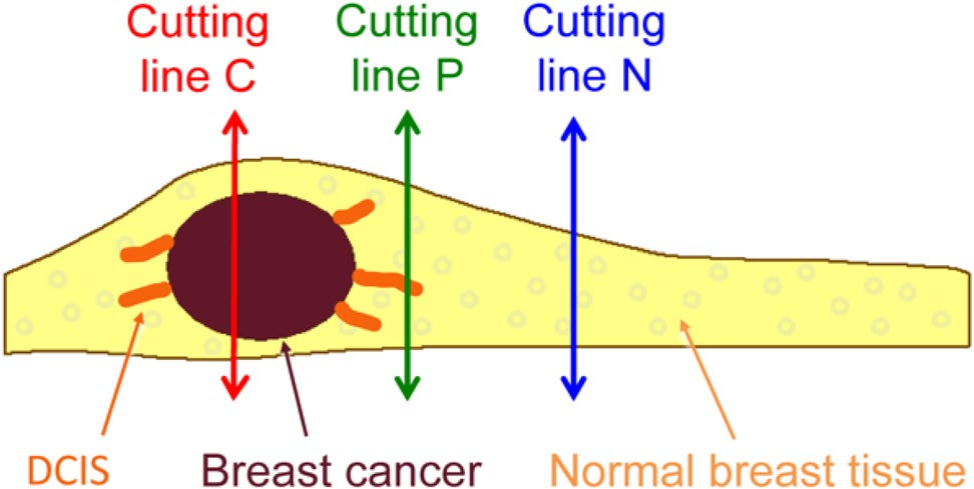
Sampling sites of the resected mammary specimens. Three samples were obtained from the surgically resected tissues. The central portion of the cancerous mass (cutting line C), a peripheral region that potentially contained non-invasive cancer cells (cutting line P), and a normal mammary tissue distant from the cancerous lesion (cutting line N) were surgically resected.

Three samples were obtained from each resected specimen: the central portion (which should contain the breast cancer tissue); the periphery (which potentially contain non-invasive cancer tissue); and the distal portion of the normal mammary tissue (Fig. 6). The samples were cut out with knives, and the surface resected by electrocautery where GGT could be thermally damaged were not examined. Each sample (3 × 3 × 3 − 22 × 22 × 6 mm in size) was first moistened with saline and then incubated with 1 mL of fluorescent probe solution (50 μM gGlu-HMRG, containing 0.5% [v/v] dimethyl sulfoxide as a co-solvent).

### Improved fluorescence measurements

Each sample was soaked in PBS solution for 0.5 − 20 min until measurement to remove blood, fat, and other extracellular materials. Each sample was then placed in a well (35 mm in diameter), and the probe solution (~ 2 mL) was poured into the well. Fluorescent imaging was started immediately after the addition of the probe solution. During imaging, the samples were soaked in a probe solution. The probe solution temperature and the atmosphere inside the instrument were equilibrated to room temperature (15–30 °C) for more than 30 min before taking measurements. The ambient temperature of sample wells was continuously recorded during the measurements. Fluorescence images were recorded using a newly developed, dedicated apparatus (Hamamatsu Photonics, Hamamatsu, Japan) with 460 nm-excitation lights, a 525 nm-emission filter, a built-in camera, a software program, and eight sample wells. Color images of the samples were recorded under visible light.

### Pathological examination

Permanent pathology specimens were prepared from the tissue samples used for fluorescence measurements. Each sample was fixed following a standard procedure, immediately after measuring the fluorescence. Hematoxylin–eosin (HE)-stained pathological sections of each sample were prepared from the same surface used for fluorescence measurement. The morphological features of the visible light images obtained during fluorescence imaging and the pathological specimens were carefully compared. Samples whose morphological features did not match were excluded from the analyses.

In the first and third steps of this study, which were performed at the Ueo Breast Cancer Hospital, a pathologist examined the HE-stained specimens.

In the second step, which was performed as a multicenter study, pairs of visible and HE-stained images were randomly numbered and provided to four pathologists without any background information. These pathologists examined the HE-stained specimens according to the guidelines of the General Rules for Clinical and Pathological Recording of Breast Cancer^29^. Each sample was classified into three categories—malignant (cancerous), not malignant (non-cancerous), or diagnosis pending. Each tissue was further categorized into one of five groups—(1) invasive cancer including invasive ductal carcinoma, invasive lobular carcinoma, and other invasive components; (2) non-invasive cancer including DCIS and lobular carcinoma in situ; (3) benign tumors including fibroadenoma, phyllodes tumor, and other benign tumors; (4) proliferative lesions including usual ductal hyperplasia, columnar cell lesions, atypical ductal hyperplasia, adenoma, papilloma, and other proliferative lesions; and (5) normal tissue. DCIS was further subclassified as low-grade DCIS and middle/high-grade DCIS. Four pathologists independently examined the samples. We considered the pathological diagnosis as being consistent when at least three pathologists classified the sample into the same category. Non-invasive cancer was further classified as low-grade DCIS if three or more pathologists interpreted the sample as low-grade.

### Evaluation of FI

The fluorescence image at each time point was subtracted from that at the start of the measurement. These subtracted images were used to evaluate the FI. The maximum FI in the subtracted image was treated as the FI of the tissue. We did not assess the area of the fluorescent region because the fluorophore generated from this probe, HMRG, is membrane-permeable^30^ and can also stain normal peripheral tissues.

### Statistical analysis

The data were collected and managed using Microsoft Excel 2011. Statistical analysis was performed using JMP software, version 13.2.1 (SAS Institute Inc., Cary, NC). Since the FI distribution did not follow a normal distribution (Fig. S2), we employed nonparametric methods, such as the Wilcoxon rank-sum test, nonparametric analysis of variance (the Kruskal–Wallis test), and the Steel–Dwass test.

## Supplementary Information

Supplementary Information.

## Data Availability

The datasets generated during and/or analyzed during the current study are available from the corresponding author on reasonable request.
