# Supplementary material for: Development of an intraoperative breast cancer margin assessment method using quantitative fluorescence measurements
Source: Sci Rep. 2022 May 20;12:8520. doi: 10.1038/s41598-022-12614-6 (PMC9122917; doi:10.1038/s41598-022-12614-6)
Supplement: Supplementary file 1 — Supplementary Information. [file 41598_2022_12614_MOESM1_ESM.docx]

**Supplementary Materials**

# Supplementary tables

## **Table S1.** Mean 5 min FIs for different breast cancer subtypes

| Subtype | N | Mean 5 min FI | S.D. | Kruskal−Wallis test |
| --- | --- | --- | --- | --- |
| Luminal A | 63 | 0.649 | 0.958 | *p* = 0.214 |
| Luminal B | 25 | 0.511 | 0.668 |  |
| Her2-positive | 8 | 1.075 | 1.425 |  |
| TN | 10 | 0.905 | 0.874 |  |

FI, Fluorescence increase; S.D., Standard deviation; TN, triple negative

## **Table S2.** Mean 5 min FIs for different clinicopathological features

| Parameter | Value | N | Mean 5 min FI | S.D. | Kruskal−Wallis test |
| --- | --- | --- | --- | --- | --- |
| T | T1 | 43 | 0.6295 | 0.9505 | *p* = 0.9522 |
|  | T2 | 38 | 0.5474 | 0.6422 |  |
|  | T3 | 5 | 0.3455 | 0.2387 |  |
|  |  |  |  |  |  |
| pN | pN0 | 61 | 0.5996 | 0.8776 | *p* = 0.3076 |
|  | pN1 | 4 | 0.2367 | 0.1876 |  |
|  | pN2 | 8 | 0.2483 | 0.2644 |  |
|  |  |  |  |  |  |
| Nuclear grade | Grade 1 | 41 | 0.7010 | 1.1419 | *p* = 0.7616 |
|  | Grade 2 | 13 | 0.6229 | 0.6498 |  |
|  | Grade 3 | 40 | 0.6509 | 0.8423 |  |
|  |  |  |  |  |  |
| Histological grade | Grade I | 16 | 0.7491 | 1.2569 | *p* = 0.9203 |
|  | Grade II | 30 | 0.8187 | 1.1318 |  |
|  | Grade III | 33 | 0.5581 | 0.6960 |  |
|  |  |  |  |  |  |
| Ly | Ly 0 | 42 | 0.8109 | 1.0380 | *p* = 0.0880 |
|  | Ly 1 | 41 | 0.3553 | 0.3572 |  |
|  | Ly 2 | 4 | 0.2289 | 0.1922 |  |
|  | Ly 3 | 1 | 0.3421 | . |  |
|  |  |  |  |  |  |
| V | V 0 | 72 | 0.5990 | 0.8321 | *p* = 0.2465 |
|  | V 1 | 11 | 0.5398 | 0.6425 |  |
|  | V 2 | 5 | 0.1634 | 0.1029 |  |
|  |  |  |  |  |  |
| Neoadjuvant chemotherapy | No | 102 | 0.6921 | 0.9451 | *p* = 0.2526 |
|  | Yes | 4 | 0.1741 | 0.0771 |  |

FI, fluorescence increase; Ly, lymphatic vessel invasion; V, blood vessel invasion; S.D., standard deviation

**Table S3.** Statistical parameters for the 5 min and 15 min FIs in each tissue type

| FI | Statistical parameters | Invasive | Non-invasive | Low-grade DCIS | Proliferative lesion | Normal |
| --- | --- | --- | --- | --- | --- | --- |
| 5 min FI | Mean | 0.724 | 0.963 | 0.385 | 0.294 | 0.145 |
|  | S.D. | 0.989 | 1.412 | 0.441 | 0.302 | 0.351 |
|  | S.E. of mean | 0.104 | 0.408 | 0.139 | 0.081 | 0.032 |
|  | 95% CI | 0.930 | 1.860 | 0.700 | 0.468 | 0.207 |
|  |  | 0.518 | 0.065 | 0.070 | 0.120 | 0.082 |
| 15 min FI | Mean | 2.209 | 2.666 | 1.207 | 1.034 | 0.475 |
|  | S.D. | 2.303 | 3.162 | 1.008 | 0.815 | 0.896 |
|  | S.E. of the mean | 0.241 | 0.913 | 0.319 | 0.218 | 0.081 |
|  | 95% CI | 2.689 | 4.676 | 1.928 | 1.504 | 0.635 |
|  |  | 1.730 | 0.657 | 0.486 | 0.563 | 0.315 |
|  | N | 91 | 12 | 10 | 14 | 123 |

FI, fluorescence increase; DCIS, ductal carcinoma *in situ*; S.D., standard deviation; S.E., standard error

**Table S4.** Results of Steel–Dwass testing among the 5 min FIs of five tissue types

| Level | Level | Difference of the mean | S.E. of the difference | Z | *P*-  value | Hodges– Lehmann estimator | 95% CI | |
| --- | --- | --- | --- | --- | --- | --- | --- | --- |
| DCIS/LCIS | Invasive | 6.178 | 9.176 | 0.673 | 0.962 | 0.080 | -0.310 | 0.504 |
| Proliferative | Low-grade DCIS | -1.250 | 3.005 | -0.416 | 0.994 | -0.033 | -0.399 | 0.303 |
| Low-grade DCIS | DCIS/LCIS | -3.208 | 2.780 | -1.154 | 0.778 | -0.148 | -1.801 | 0.337 |
| Proliferative | DCIS/LCIS | -5.625 | 3.074 | -1.830 | 0.356 | -0.209 | -1.199 | 0.150 |
| Low-grade DCIS | Invasive | -8.491 | 9.761 | -0.870 | 0.908 | -0.070 | -0.597 | 0.281 |
| Proliferative | Invasive | -15.298 | 8.567 | -1.786 | 0.382 | -0.081 | -0.470 | 0.051 |
| Normal | Low-grade DCIS | -33.250 | 12.672 | -2.624 | 0.066 | -0.123 | -0.402 | 0.004 |
| Normal | Proliferative | -35.080 | 10.934 | -3.208 | **0.012** | -0.082 | -0.287 | -0.013 |
| Normal | DCIS/LCIS | -51.631 | 11.830 | -4.365 | **0.0001** | -0.362 | -0.728 | -0.095 |
| Normal | Invasive | -78.905 | 8.562 | -9.216 | **<.0001** | -0.186 | -0.360 | -0.100 |

Z, Z-value of Steel Dwass test; CI, confidence interval; DCIS, Ductal carcinoma *in situ*; FI, Fluorescence increase; LCIS, Lobular carcinoma *in situ;* S.E., standard error

The P-values shown in bold orange are significant (*P* < 0.05)

**Table S5.** Results of Steel–Dwass testing among the 15 min FIs of five tissue types

| Level | Level | Difference of the mean | S.E. of the difference | Z | *P*-value | Hodges− Lehmann estimator | 95% CI | |
| --- | --- | --- | --- | --- | --- | --- | --- | --- |
| DCIS/LCIS | Invasive | 7.310 | 9.176 | 0.797 | 0.932 | 0.359 | -1.338 | 1.466 |
| Proliferative | Low-grade DCIS | 0.000 | 3.005 | 0.000 | 1.000 | 0.009 | -1.401 | 1.068 |
| Low-grade DCIS | DCIS/LCIS | -3.575 | 2.780 | -1.286 | 0.700 | -0.615 | -3.430 | 0.833 |
| Proliferative | DCIS/LCIS | -5.925 | 3.074 | -1.927 | 0.303 | -0.736 | -2.877 | 0.406 |
| Low-grade DCIS | Invasive | -8.935 | 9.761 | -0.915 | 0.891 | -0.282 | -2.420 | 0.655 |
| Proliferative | Invasive | -12.813 | 8.567 | -1.496 | 0.565 | -0.391 | -1.958 | 0.241 |
| Normal | Proliferative | -43.083 | 10.934 | -3.940 | **0.0008** | -0.371 | -1.218 | -0.173 |
| Normal | Low-grade DCIS | -44.712 | 12.672 | -3.528 | **0.0038** | -0.582 | -1.483 | -0.159 |
| Normal | DCIS/LCIS | -55.564 | 11.830 | -4.697 | **<.0001** | -1.262 | -2.521 | -0.594 |
| Normal | Invasive | -76.362 | 8.562 | -8.919 | **<.0001** | -0.799 | -1.463 | -0.494 |

DCIS, Ductal carcinoma in situ; FI, Fluorescence increase; LCIS, Lobular carcinoma in situ; CI, confidence interval; S.E., standard error

**Table S6.** Re-validation of the results obtained using the threshold determined in this multicenter study

|  | Cancer | Benign lesion | Normal breast tissue | Total |
| --- | --- | --- | --- | --- |
| FI + (>0.979) | 6 | 0 | 1 | 7 |
| FI ± | 14 | 3 | 17 | 34 |
| FI− (<0.061) | 0 | 0 | 5 | 5 |
| Total | 20 | 3 | 23 | 46 |

FI, Fluorescence increase

# Supplementary Figures


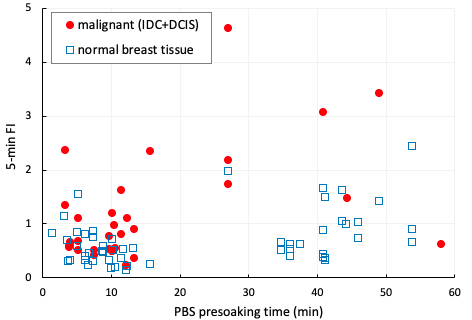


**Figure S1.** Relationship between PBS-pre-soaking time and 5-min FI

In the first-step study, 5-min FI of 31 malignant samples and 53 normal breast tissues (from 38 cases) was measured with different pre-soaking times. 5-min FIs measured after PBS pre-soaking for longer than 20 min were significantly larger than those measured after PBS pre-soaking for less than 20 min (p = 0.0014 for malignant tissues and p = 0.0015 for normal tissues by Wilcoxon rank-sum test).

**
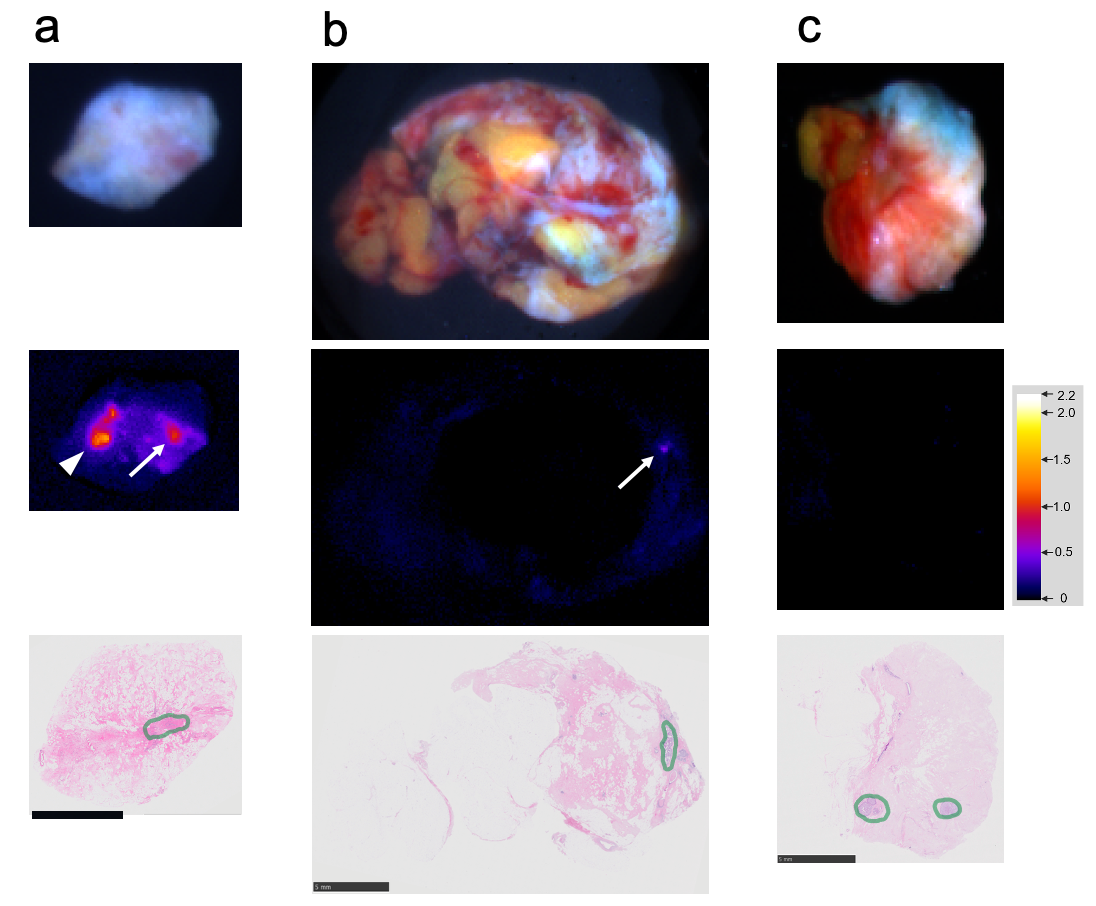
**

**Figure S2.** Examples of small lesions

Visible (upper), 5 min FI in pseudocolor (middle), and HE-stained (bottom) images of samples with small lesions. (a) Invasive ductal carcinoma, and (b, c) low-grade DCIS are indicated. White arrows on the FI images are considered to correspond to the lesions which ared indicated with green lines in each of the corresponding HE-stained images. A white arrowhead indicates a false-positive signal. Bars, 5 mm,


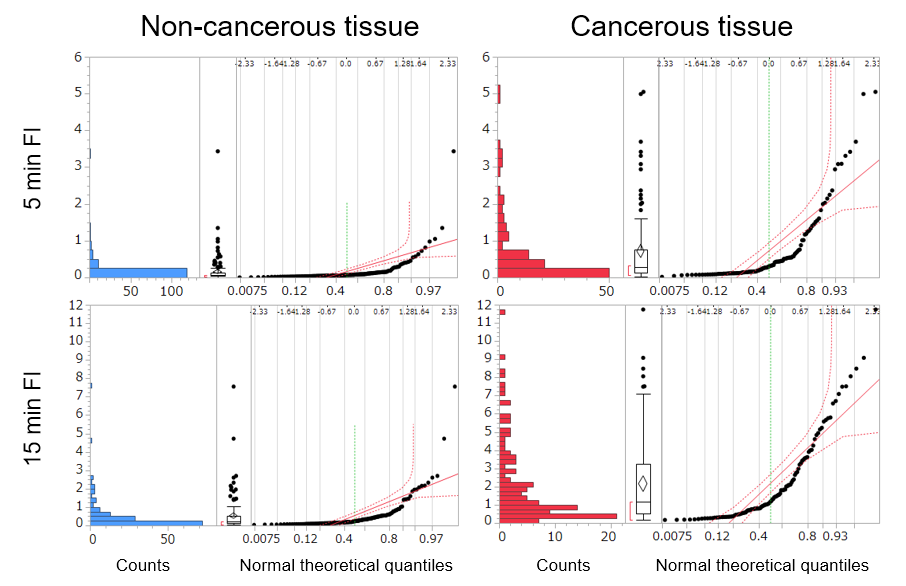


**Figure S3.** Distributions and quantile-quantile plots of the 5 min and 15 min FIs in cancerous and non-cancerous tissues

The dataset obtained in the multicenter study, including 251 sample measurements (138 non-cancerous and 113 cancerous tissues), was analyzed. The distributions and statistical parameters are indicated as histograms and box-and-whisker plots. The FIs are shown on a linear scale. In the quantile-quantile plot shown on the right side of the box-and-whisker plot, the red dotted curve indicates Lilliefors confidence bounds. FI, fluorescence increase


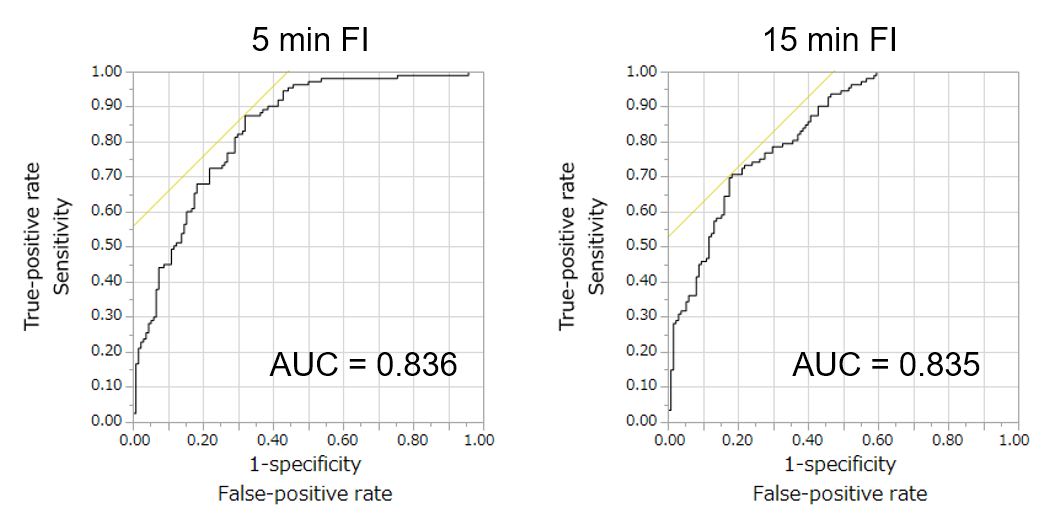


## **Figure S4.** ROC curves of the 5 min and 15 min FIs values obtained in the multicenter study

FI, fluorescence increase


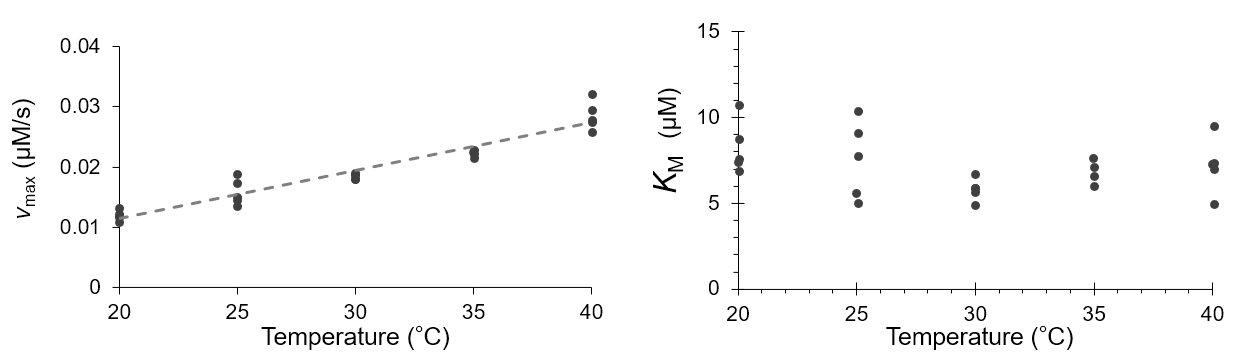
**Figure S5.** Temperature sensitivity of beef-kidney derived GGT enzyme

The Michaelis−Menten kinetic parameters were obtained from *in vitro* experiments. In these experiments, 0.11 μM GGT (γ-GT from beef kidney, 465556003, Oriental Yeast Co., Ltd.) was mixed with 0–250 μM gGlu-HMRG in PBS (pH 6.8). The fluorescence intensity for the initial 5 min was measured using a fluorescent microplate reader with 10 sec intervals (Infinite M-200 Pro, TECAN) with an excitation wavelength and emission wavelength of 480 nm and 530 nm, respectively. The mean fluorescence increase rate was calculated by the least-square fitting of the fluorescence intensity for the 5 min data. The Michaelis−Menten kinetic parameters from each set of measurements were calculated using R software (https://www.r-project.org).

##
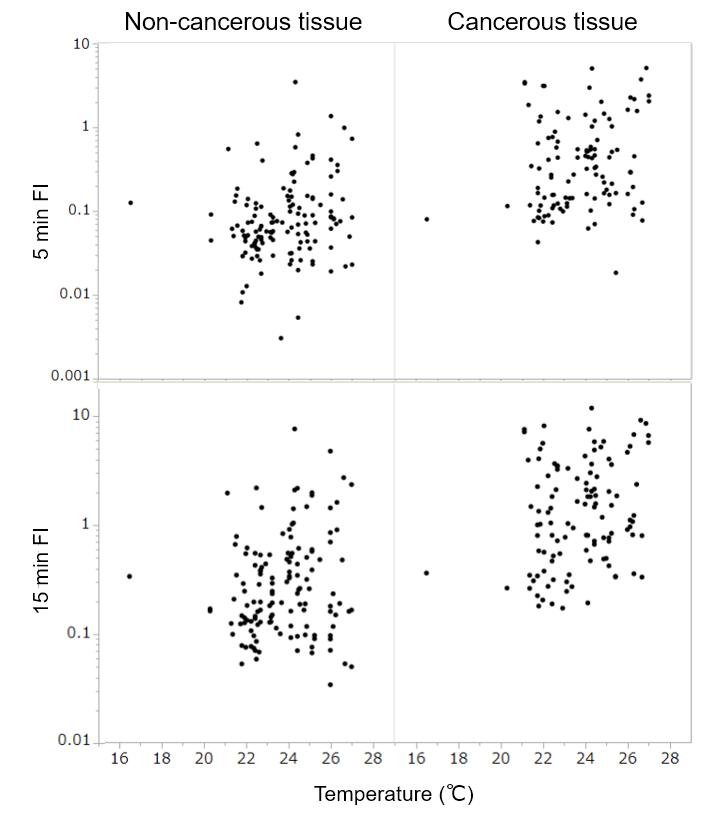


## **Figure S6.** The 5 min and 15 min FI values are plotted against the ambient temperature

FI, fluorescence increase


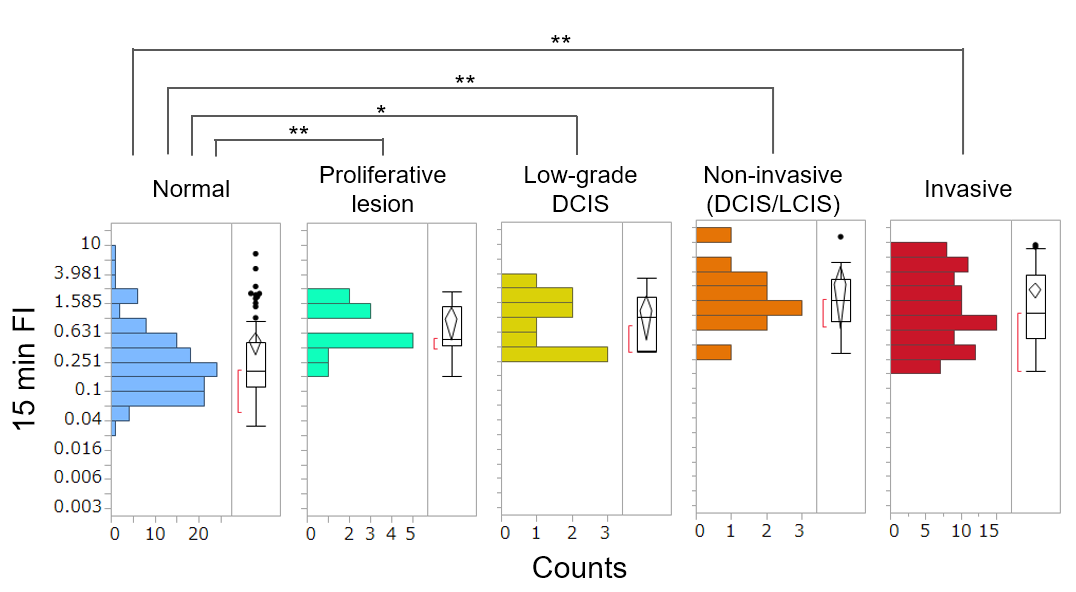


## **Figure S7.** Differences in the 15 min FI distributions between breast tissues with different lesional subtypes

The dataset obtained in the multicenter study, including 251 sample measurements, was analyzed. **p* < 0.01, ***p* < 0.001. FI*,* fluorescence increase
